# Hypothermia Reduces Mortality, Prevents the Calcium Plateau, and Is Neuroprotective Following Status Epilepticus in Rats

**DOI:** 10.3389/fneur.2018.00438

**Published:** 2018-06-11

**Authors:** Kristin F. Phillips, Laxmikant S. Deshpande, Robert J. DeLorenzo

**Affiliations:** ^1^Department of Neurology, Virginia Commonwealth University, Richmond, VA, United States; ^2^Department of Pharmacology and Toxicology, Virginia Commonwealth University, Richmond, VA, United States

**Keywords:** status epilepticus, pilocarpine, hypothermia, intra neuronal calcium levels, mortality, neuroprotection, Sprague-Dawley rats

## Abstract

Status Epilepticus (SE) is a major neurological emergency and is considered a leading cause of Acquired Epilepsy (AE). We have shown that SE produces neuronal injury and prolonged alterations in hippocampal calcium levels ([Ca^2+^]_i_) that may underlie the development of AE. Interventions preventing the SE-induced Ca^2+^ plateau could therefore prove to be beneficial in lowering the development of AE after SE. Hypothermia is used clinically to prevent neurological complications associated with Traumatic Brain Injury, cardiac arrest, and stroke. Here, we investigated whether hypothermia prevented the development of Ca^2+^ plateau following SE. SE was induced in hippocampal neuronal cultures (HNC) by exposing them to no added MgCl_2_ solution for 3 h. To terminate SE, low Mg^2+^ solution was washed off with 31°C (hypothermic) or 37°C (normothermic) physiological recording solution. [Ca^2+^]_i_ was estimated with ratiometric Fura-2 imaging. HNCs washed with hypothermic solution exhibited [Ca^2+^]_i_ ratios, which were significantly lower than ratios obtained from HNCs washed with normothermic solution. For *in vivo* SE, the rat pilocarpine (PILO) model was used. Moderate hypothermia (30–33°C) in rats was induced at 30-min post-SE using chilled ethanol spray in a cold room. Hypothermia following PILO-SE significantly reduced mortality. Hippocampal neurons isolated from hypothermia-treated PILO SE rats exhibited [Ca^2+^]_i_ ratios which were significantly lower than ratios obtained from PILO SE rats. Hypothermia also provided significant neuroprotection against SE-induced delayed hippocampal injury as characterized by decreased FluoroJade C labeling in hypothermia-treated PILO SE rats. We previously demonstrated that hypothermia reduced Ca^2+^ entry via N-methyl-D-aspartate and ryanodine receptors in HNC. Together, our studies indicate that by targeting these two receptor systems hypothermia could interfere with epileptogenesis and prove to be an effective therapeutic intervention for reducing SE-induced AE.

## Introduction

Status Epilepticus (SE) is a major clinical emergency associated with significant mortality and severe neurological morbidities amongst the survivors (1, 2). One of the major morbidities associated with survival from SE is the development of acquired epilepsy (AE) (3, 4). Epilepsy is a common neurological condition characterized by recurring spontaneous seizures. It affects approximately 1–2% of the population worldwide (1, 4). Acquired epilepsy (AE) results from a previous neurological insult such as a SE or a stroke, or a traumatic brain injury (TBI) and accounts for at least 40% of all epilepsy cases (1–4). In AE, a known cause or injury damages the brain and produces a plasticity change that leads to the development of epilepsy (5, 6). The transformation of normal brain tissue into a hyperexcitable neuronal population manifesting spontaneous recurrent epileptic discharges, or seizures, is called epileptogenesis (5–7).

It has been demonstrated in both *in vivo* and *in vitro* models of SE, stroke, and TBI that neuronal calcium (Ca^2+^) dynamics are severely altered following injury (8–10). One such alteration relevant to this study is a persistent elevation in intracellular calcium concentrations ([Ca^2+^]_i_) following SE, termed the “Ca^2+^ plateau” (8, 11, 12). The formation of the Ca^2+^ plateau has been implicated in playing a major role in the development of AE (13). Ca^2+^ is responsible for an array of cellular effects (14, 15), and alterations in a neuron's ability to regulate Ca^2+^ could substantially contribute to neuronal plasticity changes (16) that lead to epileptogenesis and subsequently AE (5, 13, 15). Therefore, preventing the rise in [Ca^2+^]_i_ immediately after SE may prevent the Ca^2+^-mediated signaling effects that lead to the development of AE. Targeting the molecular alterations observed in epileptogenesis such as elevated [Ca^2+^]_i_ may therefore offer new avenues to developing anti-epileptogenic approaches (5, 13, 15).

One such possible intervention is hypothermia. Hypothermia is used clinically to reduce neurological injury following a variety of insults including cardiac arrest (17–19), TBI (20, 21) and stroke (22, 23). Hypothermia exerts its neuroprotective effects through a variety of mechanisms including a reduction in cerebral metabolism, apoptosis, and inflammation (24, 25). One particular mechanism of interest is its ability to modulate neuronal Ca^2+^entry (26). Evidence has shown that the N-methyl-D-aspartate (NMDA) receptor mediates the majority of Ca^2+^ influx during SE (8, 11). Inhibition of the NMDA receptors during SE blocks the formation of the Ca^2+^ plateau and prevents the development of AE (5, 8). The excessive entry of Ca^2+^ into the cell during SE stimulates Ca^2+^-induced Ca^2+^ release via activation of ryanodine receptors. Evidence has suggested ryanodine receptors may be responsible for maintaining the Ca^2+^ plateau following SE (27, 28). Our lab has demonstrated in hippocampal neuronal cultures that hypothermia reduces neuronal Ca^2+^ entry through NMDA and ryanodine receptors following a high-potassium or glutamate stimulation (26). Therefore, hypothermia may prevent the formation of the Ca^2+^ plateau following SE and thus may serve as a valuable therapeutic option in preventing epileptogenesis.

This study was initiated to investigate whether hypothermia prevents the formation of the Ca^2+^ plateau following SE and decreases neuronal injury and mortality. We utilized two widely used *in vivo* (29) and *in vitro* (27) models of SE in this study. The results of this study demonstrate that hypothermia prevents the development of the Ca^2+^ plateau when administered following SE. Based on previous studies showing that blocking the Ca^2+^ plateau prevents the development of AE, the results of this study offer promising evidence that hypothermia induced following SE prevents the long lasting rise in [Ca^2+^]_i_, neuronal loss and decreases mortality and may prevent the development of AE.

## Materials and methods

All reagents were purchase from Sigma Chemical Co. (St. Louis, MO) unless otherwise specified. Cell culture media was purchased from Invitrogen (Carlsbad, CA). All animal use procedures were in strict accordance with the National Institutes of Health Guide for the Care and Use of Laboratory Animals and approved by Virginia Commonwealth University's Institutional Animal Care and Use Committee.

### Hippocampal neuronal culture preparation

Studies were conducted on primary mixed hippocampal neuronal cultures (HNC) prepared as described previously (27, 30, 31). In brief, hippocampal cells were obtained from 2-day post-natal Sprague-Dawley rats (Harlan, Frederick, MD) and plated at a density of 2.0 × 10^4^ cells/cm^2^ onto a glial support layer previously plated onto poly-L-lysine coated (0.05 mg/ml) Lab-Tek® two-well cover glass chambers (Nunc, Naperville, IL). Cultures were maintained at 37°C in a 5% CO2/95% air atmosphere and fed twice weekly with MEM enriched with N3 supplement containing 25 mM HEPES buffer (pH 7.4), 2 mM L-glutamine, 3 mM glucose, 100 μg/ml transferrin, 5 μg/ml insulin, 100 μM putrescine, 3 nM sodium selenite, 200 nM progesterone, 1 mM sodium pyruvate, 0.1% ovalbumin, 0.2 ng/ml triiodothyroxine, 0.4 ng/ml corticosterone and supplemented with a glial bed-condition media (20%). These mixed cultures were used for experiments between 15 and 21 days *in vitro* following neuronal plating.

### *In vitro* SE in hippocampal neuronal cultures and hypothermia induction

*In vitro* SE was generated using a low Mg^2+^-containing solution (27, 30, 32). Hippocampal neuronal culture media was replaced with physiological basal recording solution (pBRS) containing (in mm): 145 NaCl, 2.5 KCl, 10 HEPES, 2 CaCl2, 10 glucose, 1 MgCl2 and 0.002 glycine, pH 7.3, and osmolarity adjusted to 325 ± 5 mOsm with sucrose or pBRS without any added MgCl_2_ (referred to hereafter as low Mg^2+^). The cells were then incubated at 37°C under 5% CO2 / 95% O2 atmosphere for 3 h. During this time, neurons in low Mg^2+^ demonstrate high-frequency spiking, characterized as *in vitro* SE. Neurons treated with pBRS for 3 h served as sham-controls.

SE was terminated by replacing the low Mg^2+^ solution with either 31°C (moderate hypothermia) or 37°C (physiological temperature) pBRS. [Ca^2+^]_i_ was measured at the end of the 3 h treatment every 30 s for 20 min to get a measurement of [Ca^2+^]_i_.

### Pilocarpine model of SE and induction of moderate hypothermia *in vivo*

Sprague-Dawley male rats (Envigo, formerly Harlan) weighing 200–250 g were administered methyl scopolamine nitrate (1 mg/kg, i.p.) followed by pilocarpine nitrate (PILO) (375 mg/kg, i.p.) 30 min later. Sixty minutes after the onset of SE, rats were administered diazepam (5 mg/kg, i.p.) followed by additional diazepam injections at 3 and 5 h after the onset of SE to control seizure activity (8, 29, 33).

**Figure 1:**
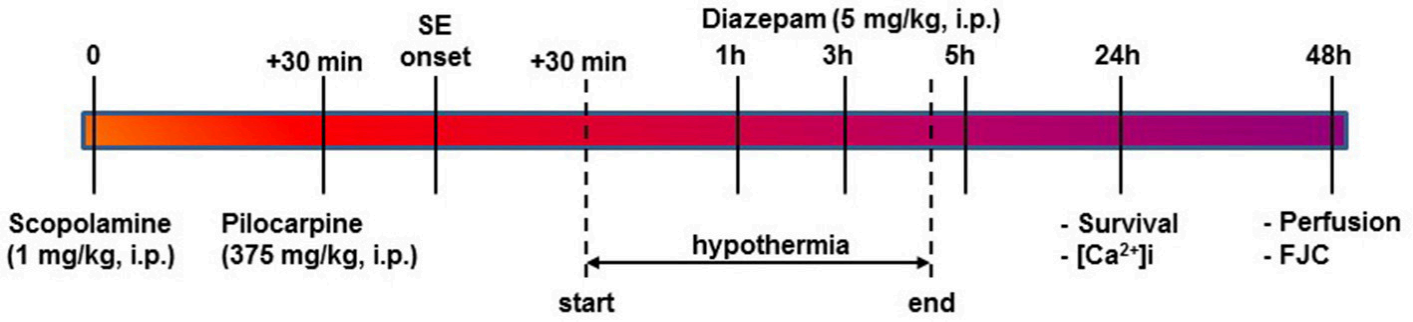
Timeline for PILO SE, induction of HYPO, and experimental endpoints. SE was induced using PILO injection and terminated with diazepam injections as per established procedures. At 30-mins into SE, moderate HYPO was induced and then maintained for 4 h using procedures described in the Materials and Methods section. At 24 h post-SE, survival and ratiometric Ca^2+^ imaging studies were conducted. At 48 h post-SE, rats were perfused and brain sections from these rats were labeled with FJC for assessing neuronal injury as described.

Moderate hypothermia was rapidly induced at 30 min post-SE onset by gently spraying rats with chilled ethanol (17°C) to speed the process of cooling. Rats were then placed in a cold room (5–8°C) for 8–10 min. Surface cooling methods were used because they are non-invasive and cost-effective. Core body temperature was determined every 2–3 min using a rectal probe (2100 Tele-thermometer; YSI, Inc., Yellow Springs, OH USA). Once core temperatures reached a 32-33°C, the rats were returned to their home cages in a room temperature environment. Core temperatures were continuously monitored and maintained between 30 and 33°C (moderate hypothermia range) with the intermittent use of ice packs and heating pads. Moderate hypothermia was maintained for 4 h at which point all the active cooling procedures were stopped and the animal was allowed to naturally rewarm to physiological temperature at room temperatures (see Figures 1, **3A**).

At 24 h post-SE, gross behavior was noted to assess recovery from SE. Rats were assigned a score of 0 or 1 on three parameters: mobility, posture, and alertness. For mobility, a score of 1 indicated rat being ambulatory, while a score of 0 indicated rat sitting in the cage, lethargic, and not moving about. For posture, a score of 1 indicated normal posture while 0 indicated a hunched posture. Alertness as indicated by being responsive to handling and approach was scored as 1 while 0 score indicated being non-responsive to handle and approach. Sum of all the scores gave the gross behavioral score and was indicative of physical recovery following SE.

### Acute isolation of hippocampal neurons

Hippocampal CA1 neurons were acutely isolated by a modification of the methods described previously (8, 10–12). The brain was rapidly dissected and placed in a 4°C chilled oxygenated (95% O_2_-5% CO_2_) artificial cerebrospinal fluid solution (aCSF) composed of (in mM) 201 sucrose, 3 KCl, 1.25 NaHPO_4_, 6 MgCl_2_, 0.2 CaCl_2_, 26 NaHCO_3_, MK-801 (1 μM) and 10 glucose (solution A). Hippocampal slices of 450 μm were cut with a vibratome sectioning system (Series 3000, Technical Products International, St. Louis, MO) and incubated for 10 min in an oxygenated medium at 34°C containing (in mM) 120 NaCl, 5 KCl, 6 MgCl_2_, 0.2 CaCl_2_, 25 glucose, and 20 PIPES, pH adjusted to 7.2 with NaOH (solution B). Slices were then treated with 8 mg/ml of Protease XXIII (Sigma Chemical Co.) in solution B for 6–8 min and then thoroughly rinsed with solution B. The CA1 region was visualized in a dark background with the help of a dissecting microscope and tissue chunks were excised. These tissue preparations were then triturated in solution B with a series of Pasteur pipettes of decreasing diameter at 4°C in the presence of acetoxymethyl (AM) form of high affinity Ca^2+^ indicator Fura-2AM (1 μM) in order to load the cells prior to [Ca^2+^]i measurements. The resulting cell suspension was then placed in the center of poly-L-lysine coated Lab-Tek® two-well cover glass chambers (Nalge-Nunc International, Naperville, IL) and immediately placed in a humidified oxygenated dark chamber at 37°C for 45 min. Fura-2 was washed off with solution B and the loaded cells were allowed to equilibrate for 15 min allowing the cellular esterase to cleave the dyes from their AM forms.

### Calcium microfluorimetry

Fura-2AM was loaded in the neurons as described above and then transferred to a heated stage (37°C) of an Olympus IX-70 inverted microscope coupled to an ultra-high-speed fluorescence imaging system (Olympus/ Perkin-Elmer). Ratio images were acquired by using alternating excitation wavelengths (340/380 nm) with a filter wheel (Sutter Instruments, Novato, CA) and Fura filter cube at 510/540 nm emissions with a dichroic mirror at 400 nm. Image pairs were captured and digitized every 15 s, and the images at each wavelength were averaged over four frames and corrected for background fluorescence by imaging a non-indicator loaded field.

In neuronal cultures, ratio measurements for individual neurons were taken at 30 s intervals for 20 min. For acutely isolated neurons, ratio measurements for individual neurons were taken at 5 s intervals for 30 s. The resulting 340/380 ratios correspond directly to the total concentration of Ca^2+^ inside the cell (8, 10–12).

### Fluoro-jade staining

Rats were sacrificed 48 h following SE. Briefly, deep anesthesia was induced in rats with ketamine/xylazine (75 mg/kg/7.5 mg/kg i.p.) mixture. Anesthetized animals were flushed transcardially with saline and perfused with 4% paraformaldehyde in a 100 mM sodium phosphate buffer (pH 7.4). Fixed brains were removed and post-fixed in 4% paraformaldehyde/phosphate buffer overnight, cryoprotected in 30% sucrose/phosphate buffer (pH 7.4) (48 h), flash frozen in isopentane and stored at −80°C until used for sectioning. Coronal sections (40 μm) were cut on a cryostat (Leica Microsystems, Wetzlar, Germany) and mounted onto microscope slides (Trubond 380; Tru Scientific LLC, Bellingham, WA). Slides were dried in a desiccant chamber at 55°C for 30 min prior to staining. Slides were first incubated in a solution of 1% NaOH in 80% ethanol for 5 min followed by hydration in a 70% ethanol and then ddH_2_O for 2 min each. Slides were then incubated in a 0.06% KMnO_4_ solution for 10 min followed by washing in ddH_2_O for 2 min. Slides were then stained in a 0.0004% Fluoro-Jade C (FJC) solution in 0.1% acetic acid for 20 min (12, 28, 34). Stained slides underwent 3x washes in ddH_2_O for 2 min each and then dried in a desiccant chamber at 55°C for 30 min. Stained slides were then cleared with xylene for 5 min and cover slipped with DPX mounting agent. Stained sections were evaluated with a fluorescent inverted microscope with a 20X (UApo 340, 0.7 n.a., water) objective and excitation/emission filters for visualization of FITC. Grayscale digital images (1,324 × 1,024, 16-bit, 1X1 binning) of FJC staining for select brain regions were acquired.

### Data analyses

For experiments performed in cell cultures, a sample size (n) of at least 6 plates per treatment group was used. Experiments in cultures were performed over several weeks so that results were representative of multiple cultures. For experiments performed in whole animal, a sample size of *n* = 15 rats per treatment group were used. For each rat, 20–30 hippocampal neurons were isolated and imaged. Individual neurons from multiple experiments were pooled to calculate average and standard error of the mean (SEM). Data is presented as mean ± SEM. To determine statistical significance between treatment groups, student's *t*-test or one-way analysis of variance (ANOVA) were used followed by Tukey *post-hoc* analysis when appropriate. A *p*-value of less than 0.05 (*p* < 0.05) was considered statistically significant. Analysis of digital images to count FJC positive cell staining was carried out with Image-J (NIH, Bethesda, MD) by thresholding for specific stain and obtaining positive cell counts using the particle analysis component (size range in pixel: 25–1,000). Digital acquisition and staining analysis parameters remained constant throughout. Statistical analysis and graphs were drawn using SigmaPlot 13 (Systat Software, San Jose, CA).

## Results

### Hypothermia blocked the development of the Ca^2+^ plateau in the HNC model of SE

**Figure 2:**
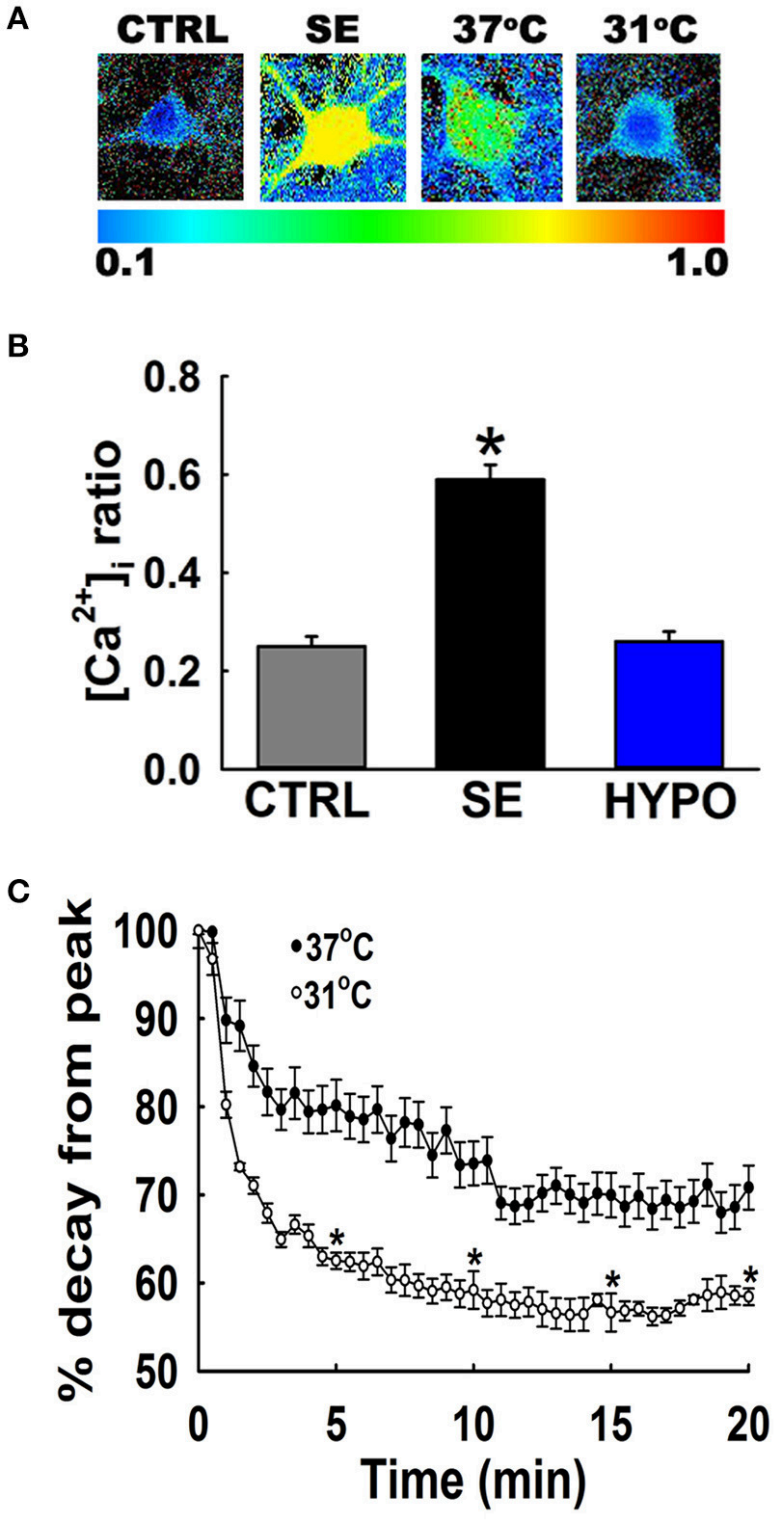
Hypothermia blocked the Ca^2+^ plateau after *in vitro* SE. **(A)** Pseudocolor ratiometric images of representative hippocampal neurons in culture from control, low Mg^2+^ SE, 37°C pBRS, and 31°C pBRS. All the ratiometric images are at the 20-min time point following respective treatments. **(B)** Fura-2 340/380 ratiometric values at the end of 20-min of treatment with hypothermic solution (31°C pBRS). **(C)** Ca^2+^ decay curves in the presence and absence of hypothermic intervention. Following 3 h of *in vitro* SE (time = 0), cells were washed with either 31°C pBRS or 37°C pBRS. 340/380 ratios were recorded every 30 s for 20 min and normalized to percent of the peak ratio observed at time = 0. *n* = 6 plates per treatment group with 40–60 neurons imaged per group. ^*^p < 0.05, Student's *t*-test for all time points after 5 min.

Figure 2A depicts ratiometric pseudocolor images of hippocampal neurons from the cultures under different temperature conditions. Fura-2 ratios were measured at 20-min following 3 h of low Mg^2+^ treatment. As illustrated in Figure 2B, hippocampal neurons washed with physiological 37°C buffer solution exhibited 340/380 ratios of 0.49 ± 0.03 compared to control ratios of 0.26 ± 0.02, indicating that [Ca^2+^]_i_ was elevated after *in vitro* SE under physiological temperature. In contrast, hypothermia treated hippocampal neurons (31°C pBRS) quickly recovered the low Mg^2+^ SE induced elevated [Ca^2+^]_i_ to baseline levels and exhibited 340/380 ratios of 0.25 ± 0.01 which were significantly lower than SE-only ratios but not significantly different from 340/380 ratios in control neurons (one-way ANOVA, *p* < 0.05).

To evaluate the dynamics of this [Ca^2+^]_i_ decay, at the end of 3 h of low Mg^2+^ treatment, [Ca^2+^]_i_ of neurons washed with either 31 or 37°C pBRS was measured over the course of 20 min. The ratio values for 31°C (hypothermia) and 37°C (normothermia) wash groups were normalized to the peak ratio (Figure 2C). After 5 min post-treatment, neurons washed with 37°C pBRS showed a slight decay in 340/380 ratios. However, cells exposed to hypothermic treatment exhibited a larger decrease in 340/380 ratios that were 62% of the peak observed at the end of *in vitro* SE. At 10 min post-treatment, the 340/380 ratio values reduced by 41 and 26% of the post-SE peak in cells washed with 31 and 37°C pBRS, respectively. At 15 min post-treatment, the ratio values fell by 44% of the post-SE peak for cells washed with 31°C pBRS and 30% of the peak for cells washed with 37°C solution. After 20 min, in the cells treated with hypothermia ratio values fell by 42% of the peak. These reductions were significantly higher than the cells washed with 37°C solution whose 340/380 ratio values fell by only 29% of the peak. Within 20 min of hypothermia treatment, [Ca^2+^]_i_ had returned to baseline ratio values of 0.25 ± 0.01 which were not significantly different from values observed in control neurons (0.26 ± 0.02). In comparison, [Ca^2+^]_i_ remained significantly elevated in cells washed with 37°C pBRS with values of 0.36 ± 0.02 (Figure 2B). There was a significant difference in the 340/380 ratio values between the two groups at each time point after 5 min of treatment (Student's *t*-test, *p* < 0.05).

### Hypothermia reduces mortality and improves recovery from SE

PILO administration induced SE in 14 out of 15 rats, with an average seizure onset latency of 17 ± 3 min. Seizure severity as indicated by Racine score was 4.0 ± 0.2. There were no deaths during the first hour of SE, at which point diazepam regime was initiated to terminate the seizures. At 24 h post-SE, 3 out of 14 rats died giving a mortality rate of 21%. The surviving rats displayed hunched posture, were less ambulatory, and were not responsive to handling or approach. Their average gross physical recovery score was 0.72 ± 0.2 (see section Materials and Methods).

**Figure 3:**
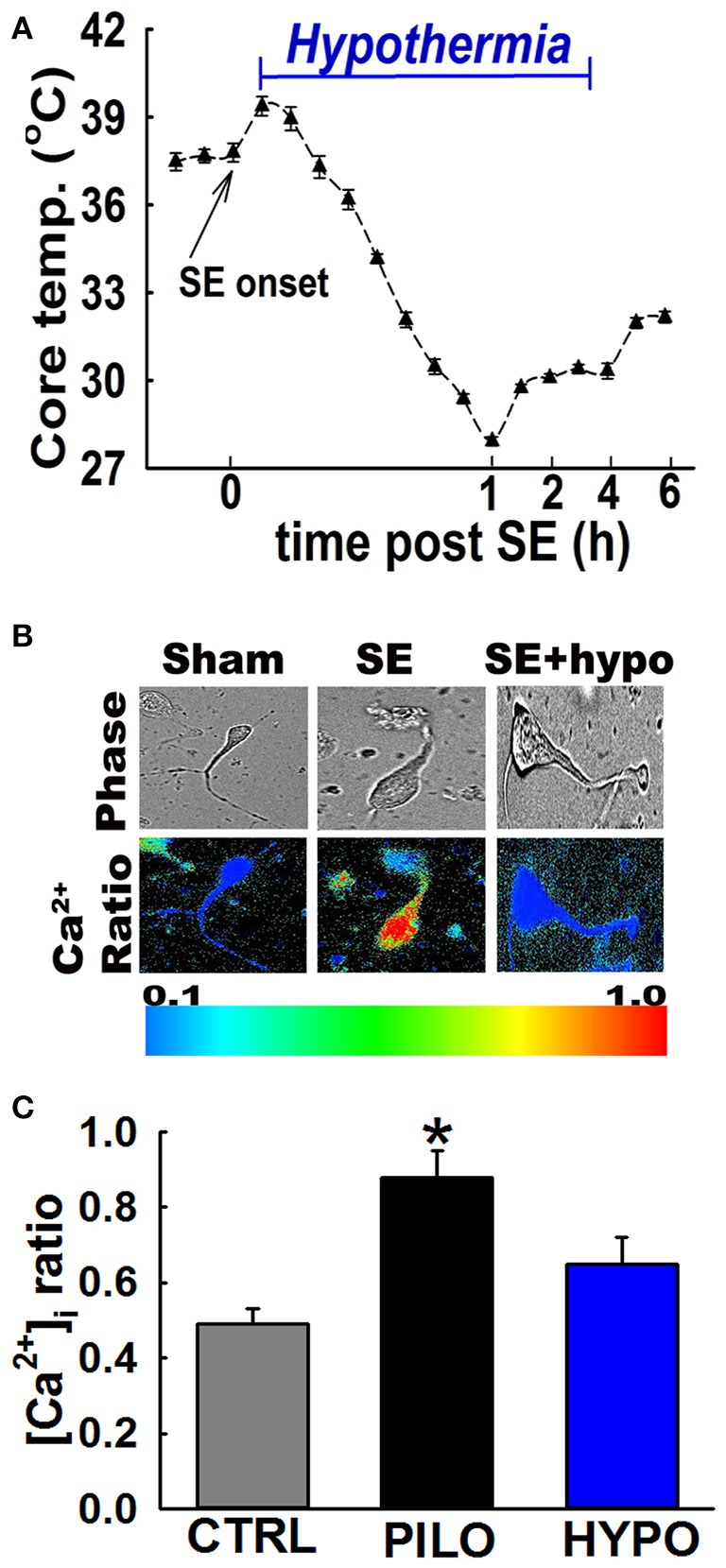
Hypothermia blocked the Ca^2+^ plateau after *in vivo* SE. **(A)** Hypothermia in rats was induced using surface cooling methods. Core body temperatures at various time points depicts rapid induction and steady maintenance of moderate hypothermia in the therapeutic range. **(B)** Pseudocolor ratiometric images of representative acutely isolated hippocampal neurons from control, PILO and hypothermia (HYPO) treated SE rats. Control and HYPO neurons had bluish color that corresponds to lower Fura-2 ratio while SE neurons had orange-red color that corresponds to higher Fura-2 ratio. **(C)** Elevated [Ca^2+^]_i_ in hippocampal neurons acutely isolated from animals at 24 h following 1 h of PILO induced SE compared to neurons from control and hypothermia (HYPO) treated rats. (^*^p < 0.001, *t*-test, *n* = 6 and 5 animals respectively). Data are represented as mean ± SEM.

In separate group of 15-rats PILO was used to induce SE using established procedures. Moderate hypothermia (31–33°C) was induced in 14-rats 30 min after PILO SE and maintained for 4 h (Figure 3A. Also see materials and methods). No differences were observed in the severity of seizures during the remainder of 30 min of SE. However, mortality rate assessed at 24 h post-SE was only 7% in hypothermia treated group. Surviving rats in the hypothermia group (*n* = 13) were alert, mobile, displayed normal posture, and were responsive to handing and approach. Fifty-percent of the hypothermia-SE survivors exhibited a complete behavioral recovery score of 3.0. Their mean gross recovery score was 2.53 ± 0.3, which was significantly higher than PILO-SE group (*n* = 13, *t*-test, *p* < 0.05).

### Hypothermia blocked the development of the Ca^2+^ plateau following SE

In order to investigate whether hypothermia was able to lower hippocampal neuronal [Ca^2+^]_i_, *in vivo*, moderate hypothermia (31–33°C) was induced in rats 30 min after PILO SE and maintained for 4 h (Figure 3A. Also see materials and methods). Hippocampal neurons were acutely isolated 24 h after SE to evaluate [Ca^2+^]_i_ using established procedures.

Figure 3B depicts ratiometric pseudocolor images of CA1 hippocampal neurons acutely isolated from rats treated with and without therapeutic hypothermia. As illustrated in Figure 3C, hippocampal neurons isolated 24 h after PILO-induced SE exhibited significant elevations in [Ca^2+^]_i_ compared to naïve controls. The average Fura-2 ratio values increased from 0.49 ± 0.04 in controls neurons to 0.88 ± 0.07 in neurons isolated from PILO-SE rats (*n* = 8 rats, *t*-test, *p* < 0.05). Thus, these results confirm our previous findings that PILO-SE results causes significant elevations in [Ca^2+^]_i_ 24 h after SE. In contrast, neurons isolated from rats treated with 4 h moderate hypothermia exhibit an average ratio value of 0.65 ± 0.07, which was significantly lower than ratios obtained from PILO-SE only rats (*n* = 12 rats, one-way ANOVA, *p* < 0.05).

### Hypothermia reduces neuronal injury following SE

**Figure 4:**
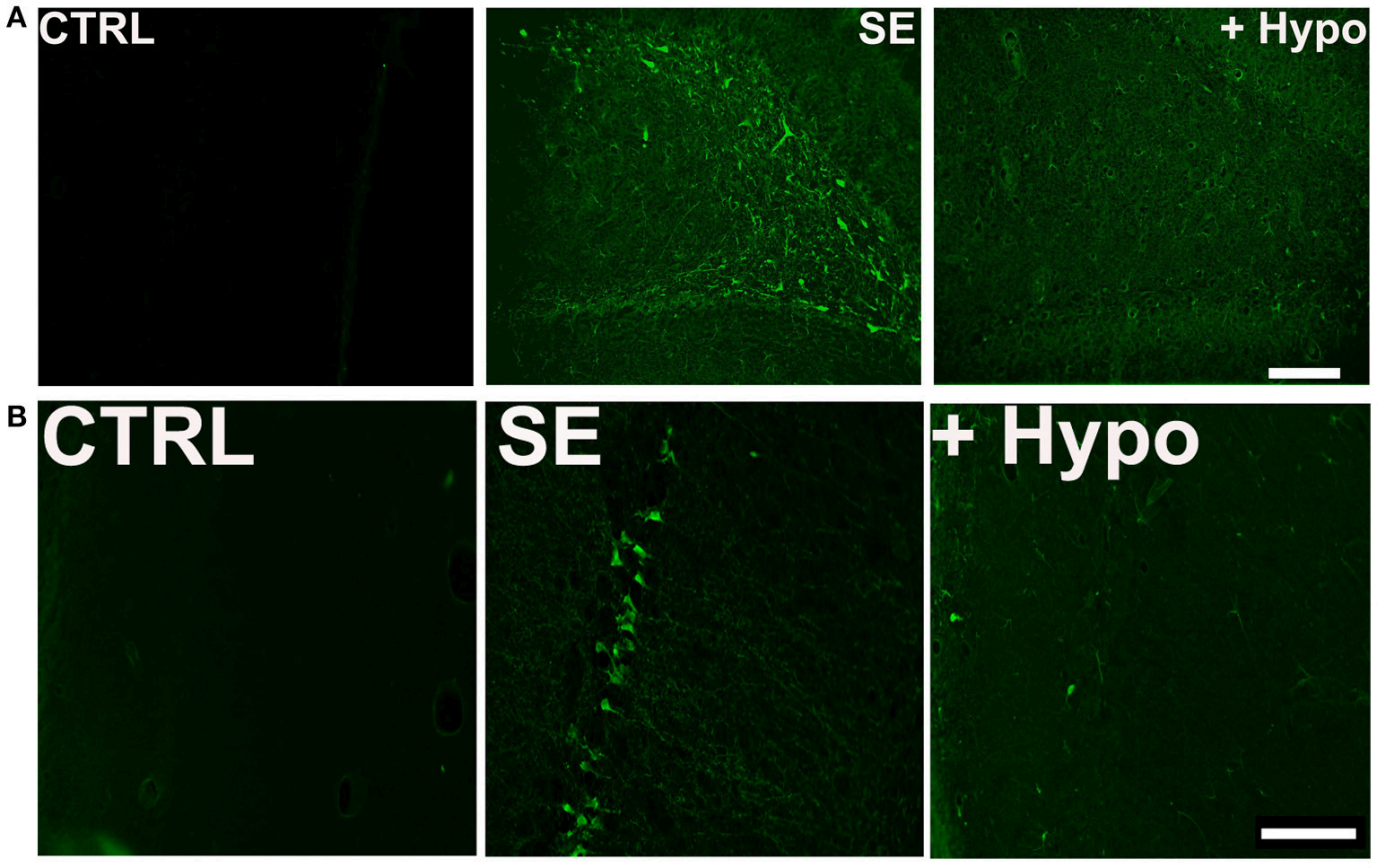
Neuroprotective effect of hypothermia. Representative photomicrographs of Fluoro-Jade C (FJC) staining in the **(A)** dentate gyrus hilus and **(B)** CA1 region of a control (CTRL), pilocarpine-SE (SE), and hypothermia (+HYPO) treated rats. Scale bars, 100 μm.

To assess neuronal injury in rats surviving SE, brain sections from SE animals treated with and without hypothermia were labeled with Fluoro-Jade C (FJC), which is an early marker of neurodegeneration (12, 28, 34). We focused on dentate-gyrus and CA1 region of the hippocampus since neurons in these regions are sensitive to SE-induced neuronal injury. Across brain regions examined, there was negligible FJC labeling in sections obtained from control rats. In contrast, after PILO-SE, within the hippocampus, FJC-positive staining was observed in the polymorphic layer and along the hilus/granule cell border of the dentate gyrus and CA1 region. An average of 8.4 ± 2.4 and 10.6 ± 3.2 FJC positive cells/ 100 μM^2^ were quantified in dentate gyrus and CA1 region respectively. Brain sections from hypothermia-treated SE rats exhibited minimal FJC staining within these regions. The quantification of the neuronal injury expressed as FJC positive cells revealed approximately a 60% reduction in FJC labeling in the dentate gyrus and CA1 region of hypothermia-SE slices with an average of 3.0 ± 1.2 and 4.4 ± 0.8 FJC positive cells/ 100 μM^2^ respectively (Figures 4A,B).

## Discussion

The results of this study demonstrate that hypothermia administered following SE not only reduces mortality but improves the recovery from SE. Hypothermia was effective at blocking the development of the Ca^2+^ plateau, and also reduced the hippocampal neuronal injury following SE.

In both the *in vivo* and *in vitro* models of SE, [Ca^2+^]_i_ remains elevated following injury, and this persistent elevation in [Ca^2+^]_i_ is believed to contribute to the pathological consequences associated with epileptogenesis. Blocking the development of the plateau has been shown to prevent the development of epilepsy in both *in vivo* and *in vitro* models of SE-induced AE (5, 8, 13, 27). The novel finding that hypothermia not only reduces mortality but also reduces elevated [Ca^2+^]_i_ and prevent the formation of the Ca^2+^ plateau offers additional information about the neuroprotective mechanisms of hypothermia as well as evidence that it could serve as an alternative intervention to pharmacological agents in preventing epileptogenesis after SE. This study has enormous translational potential for clinical application since hypothermia is already used in hospitals and ambulances settings to provide neuroprotection following cardiac arrest and hypoxia (18, 19, 23, 35, 36).

It has been demonstrated in the PILO model of SE-induced AE that SE causes a significant rise in [Ca^2+^]_i_ and these elevations persist in animals that eventually develop epilepsy (8, 37), which suggests that the Ca^2+^ plateau is involved the progression of epileptogenesis to the epileptic phenotype (5, 13). Ca^2+^ is a ubiquitous second messenger involved in a variety of cellular processes including neurotransmitter release and protein transcription, as well as long-term potentiation (14, 16, 38). Excessive elevations in [Ca^2+^]_i_ activate several downstream effectors leading to plasticity changes and eventually activation neuronal death pathways (38–41). Therefore, modulating the rise in [Ca^2+^]_i_ may be effective in blocking the Ca^2+^-mediated cascade that leads to plasticity changes associated with epileptogenesis, thus preventing epilepsy.

Blocking the formation of the Ca^2+^ plateau with the use of NMDA receptor antagonists prior to and during SE prevents the development of AE (8, 11, 27), further suggesting that elevations in [Ca^2+^]_i_ are involved in the pathophysiology associated with epileptogenesis. However, NMDA antagonists are ineffective at preventing AE if administered after the injury (8, 11, 27). Currently, there are no anti-epileptogenic drugs that can be administered following a neurological insult (42). Thus, it is important to develop a therapy that can block epileptogenesis when administered post-SE injury (6).

There are several postulated mechanisms for neuroprotective actions of hypothermic interventions (24, 25). Hypothermia is known to reduce cerebral metabolism and improve post-ischemic glucose utilization (43). Studies have reported beneficial effects of hypothermia on cellular redox regulation. For example, under ischemia/reperfusion conditions, therapeutic hypothermia suppresses enhanced oxidative stress and enhances anti-oxidative potency (44). Hypothermia is also reported to inhibit several aspects of apoptosis including increased production of anti-apoptotic protein Bcl2 (45) and inhibition of cytochrome C (46). In addition, hypothermia has been shown to produce an anti-inflammatory effect in the brain. Amongst other mechanisms, hypothermia suppresses microglial activation (47) and inhibits inflammatory transcription factor nuclear factor kappa B (48). Hypothermia also reduces excitotoxic insult by attenuating glutamate release following ischemia (49). NMDA receptor-mediated Ca^2+^ entry has been shown to play a major role in excitotoxic neuronal injury and cell death (11, 12, 50). We have shown that hypothermia reduces Ca^2+^ entry through NMDA receptors (26), and this could be an important mechanism for the strong neuroprotective effects observed after SE following hypothermia. Thus, the mechanisms underlying the neuroprotective effects of hypothermia are multifactorial. Although they have different inciting injuries, stroke, TBI, and SE share the common pathology of elevated [Ca^2+^]_i_, and subsequent neuronal loss (5). Therefore, it stands to reason that hypothermia would provide similar neuroprotection after SE as it does with stroke and TBI. Indeed, hypothermia has been shown to reduce neurological injuries associated with out-of-hospital cardiac arrests (17–19), stroke (22, 23), and TBI (20, 21).

Hypothermia could be a promising therapeutic alternative for modifying epileptogenesis and AE outcomes after SE. It has been shown to have anticonvulsant properties and has been used to treat refractory SE (51, 52). The mechanism by which hypothermia prevents the persistent rise in [Ca^2+^]_i_ is most likely mediated by its ability to modulate Ca^2+^ influx into the cell via NMDA and ryanodine receptors. However, NMDA receptor activation is not responsible for Ca^2+^ influx post-SE. At this point, the influx of Ca^2+^ following SE recruits ryanodine receptors, resulting in enhanced Ca^2+^-induced Ca^2+^ release. Ryanodine receptor activation is believed to be involved in maintaining the Ca^2+^ plateau in SE (12, 27, 28). Interestingly, hypothermia is also effective at reducing Ca^2+^ entry via ryanodine receptors (26), thus preventing the formation of the Ca^2+^ plateau. Indeed, as shown in this study, hypothermia administered 30 min post-SE blocked the protracted hippocampal Ca^2+^ elevations, and extended significant neuroprotection in hippocampus following SE.

The results of this study have important clinical implications. The neuroprotective mechanisms of hypothermia are complex and not completely understood. These findings offer additional insight into how hypothermia protects against neurological injury. In addition, the results suggest that hypothermia may prevent epileptogenesis by blocking the formation of the Ca^2+^ plateau. Further research to investigate the long-term effects of hypothermia following SE are necessary to evaluate the therapeutic potential of hypothermia as an anti-epileptogenic intervention.

## Author contributions

RD conceptualized the study. KP and LD designed and conducted the experiments. KP, LD, and RD analyzed the data and drafted the manuscript the paper.

### Conflict of interest statement

The authors declare that the research was conducted in the absence of any commercial or financial relationships that could be construed as a potential conflict of interest.
